# Chiral Monolithic Silica-Based HPLC Columns for Enantiomeric Separation and Determination: Functionalization of Chiral Selector and Recognition of Selector-Selectand Interaction

**DOI:** 10.3390/molecules26175241

**Published:** 2021-08-29

**Authors:** Mufarreh Asmari, Xiaoyu Wang, Natalia Casado, Marjan Piponski, Sergiy Kovalenko, Liliya Logoyda, Rasha Sayed Hanafi, Sami El Deeb

**Affiliations:** 1 College of Pharmacy, King Khalid University, Abha 62529, Saudi Arabia; masmri@kku.edu.sa; 2 Department of Chemical and Biomolecular Engineering, University of Notre Dame, Notre Dame, IN 46556, USA; xwang58@nd.edu; 3 Departamento de Tecnología Química y Ambiental, E.S.C.E.T, Universidad Rey Juan Carlos, C/Tulipán s/n, Móstoles, 28933 Madrid, Spain; natalia.casado@urjc.es; 4 Replek Farm Ltd., st. Kozle 188, 1000 Skopje, North Macedonia; piponski99@gmail.com; 5 Department of Organic and Bioorganic Chemistry, Zaporizhzhia State Medical University, Maiakovskyi avenue 26, 69035 Zaporizhzhia, Ukraine; kovalenkosergiy@gmail.com; 6 Department of Pharmaceutical Chemistry, I. Horbachevsky Ternopil National Medical University, Maidan Voli 1, 46001 Ternopil, Ukraine; logojda@tdmu.edu.ua; 7 Department of Pharmaceutical Chemistry, Faculty of Pharmacy and Biotechnology, German University in Cairo, Cairo 11835, Egypt; rasha.hanafi@guc.edu.eg; 8 Institute of Medicinal and Pharmaceutical Chemistry, Technische Universität Braunschweig, 38106 Braunschweig, Germany

**Keywords:** chiral chromatography, monolithic silica, chiral selector, immobilization, enantiomers, enantiomeric separation, enantiomeric impurity, molecular modeling

## Abstract

This review draws attention to the use of chiral monolithic silica HPLC columns for the enantiomeric separation and determination of chiral compounds. Properties and advantages of monolithic silica HPLC columns are also highlighted in comparison to conventional particle-packed, fused-core, and sub-2-µm HPLC columns. Nano-LC capillary monolithic silica columns as well as polymeric-based and hybrid-based monolithic columns are also demonstrated to show good enantioresolution abilities. Methods for introducing the chiral selector into the monolithic silica column in the form of mobile phase additive, by encapsulation and surface coating, or by covalent functionalization are described. The application of molecular modeling methods to elucidate the selector–selectand interaction is discussed. An application for enantiomeric impurity determination is also considered.

## 1. Introduction

The separation and identification of chiral compounds is a relevant issue in several areas of science like the pharmaceutical, agrochemical or food analysis due to the important rule of chirality for human and the environment. As a result of the dramatic case of the chiral drug thalidomide, it was revealed that enantiomers can exhibit different biological, pharmacokinetic, and pharmacodynamic properties in a chiral environment as the human body, showing different stereospecific recognition to receptors and active sites [1]. Consequently, the biological or pharmacological activity of chiral compounds is generally associated with only one of the enantiomers, while the other can be inactive, less active, toxic or show a completely different activity [2]. For instance, the R-enantiomer of the drug lacosamide shows antiepileptic activity, while the *S*-configuration is inactive [3]; *R*-duloxetine presents less antidepressant power than its *S*-enantiomer [4]; the famous thalidomide, whose R-enantiomer has sedative and antiemetic properties but its *S*-enantiomer is teratogenic and toxic [5]; or fluoxetine, whose *S*-enantiomer is effective against migraines while its R-configuration is useful against anxiety and sexual dysfunction [6]. For this reason, it is of the utmost importance to commercialize drugs as enantiomerically pure formulations to avoid exposing the organism to inactive or even harmful compounds. Likewise, the occurrence of inactive or less active stereoisomers in agrochemicals contributes to increased environmental pollution without any benefit on the desired action [7]. On the other hand, pure enantiomers are also required in the preparation of amino acid-based food supplements and additives, as the use of D-enantiomers (*R*-configuration) is forbidden in the food industry [8]. Consequently, the preference for pure enantiomer formulations is evident. In the case of drugs, during the last decades, the trend to commercialize drugs as enantiomerically pure formulations has risen significantly [1,7]. In fact, this increase has been partly encouraged by the market strategy so-called racemic switch, which enable the companies to achieve a patent on a new-single pure enantiomer formulation of a drug that was first sold as racemate [4]. Nonetheless, many drugs are still marketed as racemic mixtures, even though there is only one active enantiomer. In fact, currently, more than 60% of the drugs commercialized are chiral, and approximately an 88% of them are sold in their racemic form [2]. These racemic formulations should only be administered when enantiomers show complementary biological activity. Moreover, even when both enantiomers are active, it is still more advantageous to use single pure enantiomeric formulations, because lower doses can be used, there is higher safety margin, lower interindividual variability, as well as less drug interactions and side effects [1,2]. 

Therefore, the growing demand for pure enantiomer formulations requires the development of sensitive analytical methodologies that enable the detection and differentiation of enantiomers and the control of the enantiomeric purity of the marketed products. In this sense, liquid chromatography (HPLC) has been widely applied for the analysis of chiral compounds due to its multiple advantages and suitability to achieve the separation of enantiomers.

In contrast to conventional liquid chromatography with particle packed columns, highly efficient fast separation is usually desired in different fields including but not limited to pharmaceutical, biological, food and environmental analysis. Fast efficient separation in chromatography is conducted by three competing approaches; ultrahigh pressure HPLC, core-shell and monoliths. 

Ultrahigh pressure HPLC is based on the use of small sub-2-µm non-porous particle packed columns that can provide large surface area than the classical particle packed stationary phase for more efficient separation thus allowing the use of shorter columns with equivalent resolution to save the analysis time. The accompanied high back pressure of the small particle packed columns is overcoming by running the column in an ultrahigh pressure HPLC instrument that can resist high back pressure of up to 15,000 psi or 10,000 psi for longer column lifetimes. This has been achieved by the introduction of UHPLC system to run the chromatographic process with the sub-2-µm particle packed columns [9]. 

The second approach to achieve highly efficient fast chromatographic separation depends on changing the type of packed stationary phase particles inside the columns. Instead of using solid sphere particles, a core-shell particle (superficially porous particles) is used. The core shell involves the use of a solid core particles coated with a thin of stationary phase. A short diffusion path length of the shell improves mass transfer and the separation efficiency, as well as it ensures a reduced plate height [10,11]. The smaller eddy dispersion time also contributes to better separation efficiency [12]. However, lower sample load capacity is a common disadvantage and possible weaker retention would be obtained. 

The third approach is the use of monolithic HPLC columns, which are columns of stationary phase contain continuous porous material for chromatographic separation. Monolithic silica was introduced first by Nakanishi et al. [13,14] in the early 1990s fabricated by sol-gel phase process transformation. Tanaka et al. [15,16] described the fabrication process to obtain a monolithic column. Based on the filling material, three types of monolithic column are known, i.e., polymeric-based monolithic columns, hybrid-monolithic columns, and monolithic-silica columns. Polymeric-based columns are particularly efficient in separating large molecules such as proteins and are more used in capillary rather than conventional HPLC columns, mostly for the separation or functionalization of large biomolecules, such as enzymes, proteins, and antibodies [17,18]. Hybrid monolithic columns of a mixed modes showed also successful applications [19,20,21]. Nano-LC monolithic capillary columns are also used for enantioseparation. Different types of chiral selectors have been immobilized into monolithic capillary columns either to silica-based [22,23,24] or to polymeric-based [25] or to hybrid monolithic [26] capillary columns for chiral discrimination. Examples of immobilized chiral selectors include cyclodextrins as well as derivatives of cellulose, amylose, and macrocyclic antibiotics, among others [25,27,28]. While chiral selector immobilized into polymeric-based monolithic capillary columns are more reproducible [25,29], silica-based monolithic silica capillary columns show better enantioresolution power [28]. 

In 2000 monolithic-silica columns were commercialized by two companies Merck KGaA (Darmstadt, Germany) and Phenomenex (Torrance, CA, USA). Merck started first by introducing Chromolith®Performance RP-18e column 4.6 × 100 mm monolithic silica rods enclosed in polyetheretherketone (PEEK) column body. Different modes (Si, RP8, RP18, NH2) then appeared and are these currently commercialized [30]. Phenomenex also produced monolithic silica columns commercially as Onyx^TM^ monolithic available in C8, C18. The second-generation monolithic (Chromolith^®^ HighResolution RP-18e columns (100 × 4.6 mm) followed the first by Merck in 2011. It shows improved radial pore distribution and reduced the size of macropores to correspond in performance to sub-2-µm conventional particle packed columns with better peak symmetry than the first generation. Reduced theoretical plate heights are usually obtained with the second-generation monolithic columns. Higher backpressure is usually obtained with second generation compared to first generation monolithic columns but still lower than that of a conventional particle-packed column [31,32]. Macropores of a second-generation column has a diameter of 1.15 µm diameter and is claimed to give a column efficiency exceeding 140,000 plates/meter. The smaller eddy dispersion obtained by smaller macropores size reduces band broadening compared to that possible with the first-generation skeleton. The separation performance of HighResolution is claimed to correspond to sub-3-µm total porous or 2.7-µm core-shell columns [33]. Monolithic silica HPLC columns are currently available in different i.d. (2, 3, 4.6 mm) and different lengths (25, 50, 100, 150 mm). Capillary format monolithic (C8 and C18) of i.d. 50, 100 and 200 µm and 5, 15 and 30 cm lengths are also available.

This review focused on the use of monolithic silica based HPLC columns for chiral separation and determination of small chiral compounds. Nano-LC nonolithic capillary columns for enantioseparation are also reported. Polymeric and hybrid monolithic columns are also showing some applications for chiral separation. Accordingly, this review is devoted to chiral monolithic silica stationary phases which can be either added to the mobile phase as CMPA or coated or functionalized to the surface of the monolithic silica [34,35,36]. The application of molecular modeling to elucidate the chiral recognition is discussed. The review aims to enhance the application of monolithic columns particularly the silica-based type to the enantioseparation of chiral compounds to profit from their advantages.

## 2. Monolithic Silica HPLC Columns for Chiral Analysis

### 2.1. Propoerties of Monolithic-Silica Stationary Phase

Monolithic silica stationary phase has a bimodal pore structure of macropores (through-pores) forming a three-dimensional network of a one-piece continuous rod. The macropores allow for convective flow of the mobile phase and are responsible for the high permeability of the monolithic silica columns. The high permeability and the high stability of the silica structure allows the use of a high flow rate and thus a reduction in the analysis time. The other pores are located in the interconnection of the network and are referred to as mesopores. Mesopores provide larger surface and are mainly responsible for an enhanced mass transfer. Those smaller pores also decrease the height equivalent to theoretical plate (HETP) and increase the plate number thus a better column efficiency and performance is obtained. Compared to particle packed columns, monolithic silica columns usually have same selectivity, no frit, larger surface area, and result in higher resolution with less peak tailing. Monolithic columns also usually show better van Deemter curves (less drop in plate height at high mobile phase velocity) compared to particulate columns of 3.5 and 5 µm. On the other hand, sub-3 µm particle columns can give slightly better van Deemter curves compared with sub-2 µm particle columns [37,38]. Moreover, monolithic columns are claimed to be more stable than particle packed columns due to the rigid one-piece skeleton. The limited pH and temperature resistance of conventional packed silica stationary phase are inherited by monolithic silica stationary phase. Flow rates up to 10 mL/min are possible with monolithic silica HPLC column to result in a very short analysis time with an acceptable rise in backpressure that does not exceed the instrument limit (e.g., 200 bar for a 100 × 4.6 mm column) and a minimum decrease of resolution. The low mass transfer resistance compared to conventional particle-packed column decrease the negative impact on resolution at high flow rate. It is worth noting that the acquisition rate of the detector should be re-adjusted to 200 ms or less to compensate the fast passage of the compounds through the detection cell at the high flow rate [33,39,40,41]. As with particle-packed columns, monolithic columns are compatible for LC-MS and LC-MS/MS with or without post column split [42,43]. Many basic compounds showed better peak shape on monolithic silica columns than on particle-packed columns [44]. It is noteworthy to mention that; monolithic columns show lower loadability compared to conventional particle packed columns of the same size. This is due to the lower density of monolithic silica columns compared with the same size conventional particle packed column. However, the injection amounts for analytical purposes are usually less than the maximum loadability [45]. Thus, monolithic columns outperform the conventional columns in HPLC applications and can generate column efficiency of several thousand theoretical plates per meter [46]. Monolithic silica columns are also suitable for bioanalysis because of the low clogging susceptibility of the pores due to the high porosity. Lower column blockage possibility made it possible even to directly inject a diluted plasma sample or plasma extract for bioanalysis. This will generally reduce the need for sample pretreatment and a longer lifetime of the column [42]. Due to the high porosity and high permeability of monolithic columns, the coupling of two or more columns is possible using available column couplers. More plate numbers will be gained with some possible loss of plate numbers due to coupling. This can be used to increase the separation efficiency when analyzing a complex mixture.

### 2.2. Monolithic Column Coupling

Coupling of two or more monolithic silica columns can also help in obtaining chromatographic fingerprint of a complex mixture. It can also be used to conduct two- or multi-dimensional HPLC separation by connecting two columns of different chemistry (e.g., reversed-phase with ion-exchange) and thus different retention mechanisms, but with a complementary selectivity. In chiral separation, this could be utilized to connect two or more chiral-functionalized columns, each having a different chiral selector bounded on its surface. Dual or multidimensional system would help to carry on an orthogonal collaborative enantiodiscrimination power using various combinations of chiral selectors each with a different chiral recognition mechanism. Thereby, the possible unwanted interaction between the chiral selectors is also hindered in contrast to mixing them and adding them as CMPA. The increase in analysis time due to column coupling and longer total columns length could be compensated by flow-programming [47]. Two dimensional (2D-HPLC) mostly of chiral achiral columns with or without a switching valve is showing good success in chiral separation [48,49].

### 2.3. Flow Programming Elution

Alternative to gradient elution, flow programming can be used with monolithic silica columns to reduce the analysis time in a chromatogram with late eluting peak. No re-equilibrium time is required between runs, in contrast to gradient elution, because the mobile phase composition remains constant. This is not possible with conventional columns due to the increase in backpressure that will exceed the maximum allowable backpressure limit of the conventional HPLC instrument [50,51]. The larger surface coverage of monolithic silica compared to conventional particle-packed columns with chiral selector either by coating through physical adsorption or by immobilization ensures higher yield of attached chiral selector and thus larger loading capacity and better enantioseparation [52]. Good precision regarding peak area and retention time was reported for the commercial monolithic columns in addition to good column-to-column reproducibility [38]. Due to possible rapid column flushing, re-equilibrium, use of high flow-rate, column coupling and possible use of flow programming an updated method development strategy was mandatory. El Deeb et al. [53] proposed a clear method development strategy (update of Snyder’s method) to develop a method using monolithic silica HPLC columns describing all the steps for different compounds chemistry. Different successful chromatographic methods have been developed using monolithic silica columns also coupled to quadrupole mass spectrometry [43,54,55].

### 2.4. Introducing Chiral Selector into the Monolithic Silica Stationary Phase

For the three major types of matrices composing monolithic columns namely: silica-based (most common), organic polymer-based, and organic-silica hybrid monolithic, many chiral selectors (CS) for chromatographic techniques [56] and capillary electrophoretic techniques [57] are available and are continuously optimized and improved by researchers in the field to achieve maximum reproducible enantioselectivity as well as column mechanical stability. Those CS can be classified into nine families based on their chemistry, namely cyclodextrins, protein and glycoproteins, polysaccharides, molecularly imprinted polymers, macrocyclic antibiotic, chiral ligand exchange, chiral ion exchange, donor-acceptor type, and crown ethers based.

The most common and extensively studied among these are cyclodextrin derivative-based [58], as well as protein and glycoprotein-based (human serum albumin, trypsin, pepsin, Alpha1- acid glycoprotein) [59] which suffer from insufficient affordability and low immobilization efficiency on the monolith. In attempt to widen application scope in CEC of protein CS, mixed functionalization of the protein with the third family: polysaccharide CS takes place where co-immobilizing of HSA and cellulase on the monolith is reported [60]. This third family of polysaccharide-based (cellulose and amylose derivatives) [61] is mostly used in LC applications where their derivatization promotes their applicability from only polar to less polar analytes [62]. In addition, to improve mechanical stability and enantioselectivity, switching from purely silica as monolithic supports for polysaccharide immobilization, zirconia-based and organic-silica hybrid monoliths have proven to be efficient alternatives. Molecularly imprinted polymer (MIP)- based represent the fourth family of CS that is subject to a growing number of studies to enhance its applicability and overcome its problems of morphology change when exposed to different mobile phases, especially when the monolith is not silica-based [63]. To stabilize binding sites and improve molecular recognition and enantioselectivity of the MIP, usage of molecular crowding agent (ex: polymethyl methacrylate) has been adopted [64]. The versatile macrocyclic antibiotic-based CS widely used in classic particulate columns has also been a player in chiral selectivity of monoliths and represents the fifth family. Even though Vancomycin was one of the earliest approached macrocyclic as monolith CS, it was seldom used in HPLC, more commonly on CE with norvancomycin [65] especially when Zirconia-based monoliths improved the CS stability [66]. Yet, other macrocyclic such as colistin sulfate have been assessed in reversed phase mode successfully with α- and β-blockers, anti-inflammatory and antifungal drugs, norepinephrine-dopamine reuptake inhibitors, catecholamines, sedative hypnotics, antihistaminics, anticancer, and antiarrhythmic drugs [67]. In sixth position comes the chiral ligand exchange CS type that is simple and inexpensive to form whether on silica-based or polymer-based matrix [68]. It relies on formation of ternary metal complexes between chiral compounds and the CS providing high efficiency and reproducibility for separation of amino and hydroxy acids derivatives. Chiral charged analytes were successfully resolved on the seventh family of CS, namely chiral ion exchange type [69], that found most of their applicability with zwitterionic cinchona-based CS in CEC mode [70]. The donor-acceptor type (Brush/Pirkle type) is a CS that depends on derivatives of small molecules such as valine, leucine, and proline as the CS of the monolith. Interestingly, polyproline chiral monolithic columns were reported to provide better performance in SFC than classic particulate column counterparts [71]. The CS monoliths are capable of enantioresolving a multitude of chiral drugs with different chemistries, including amino acids, small peptides, basic and acidic drugs, such as a- and β-blockers, NSAIDs, barbituric acid derivatives, antifungal drugs, dopamine antagonists, sedative hypnotics, and antihistamines [72], though mostly in laboratory prepared mixtures and rarely on real samples due to the currently lasting superiority of particulate chiral columns over chiral monoliths in terms of efficiency, reproducibility and enantioresolution. The last family of CS introduced to monolithic columns is the crown ethers-based, which were used for the successful enantioseparation of polycyclic aromatic hydrocarbons, benzenediols, carbamate pesticides and steroids [73] and ɑ-amino acids [74].

**Figure 1:**
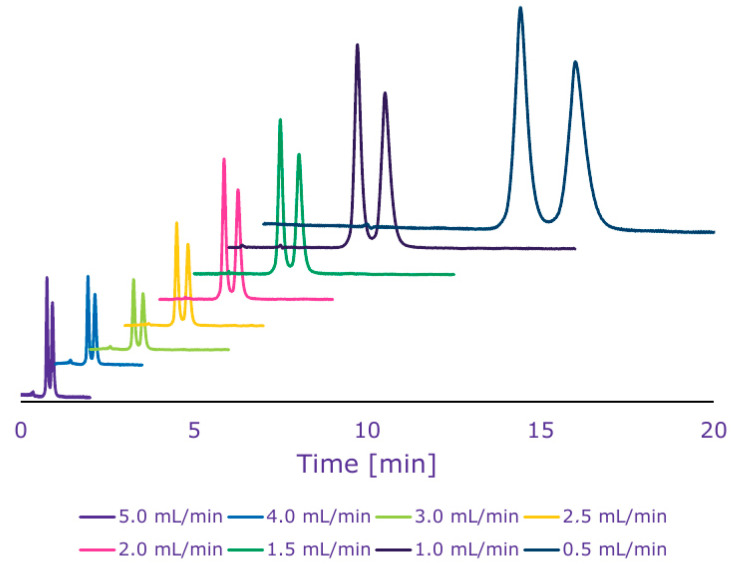
Representative chromatograms showing the separation of terbutaline enantiomers at different flow rates using cellulase immobilized epoxy monolithic silica columns. Reprinted with permission from Merck KGaA, Darmstadt, Germany.

Successful introduction of chiral selectors to monolithic columns depends primarily on the selection of the most appropriate assembly approach to ensure that a proper amount of the CS is attached to the monolith to provide optimum enantioselectivity, in addition to assuring quality of repeatability, mechanical and chemical stability, maximum surface area of the CS, and maximum column plate number. A large repertoire of methods is available, including immobilization, physical coating, adsorption, attachment, encapsulation, binding, covalent bonding, particle fixing, and on-column derivatization, where in this later case functionalization of the monolith surface is first performed to receive the CS via a reaction between the silica’s silanol groups and a suitable reagent with the desired functional moiety that would later accept the CS. For silica-based monoliths, physical adsorption is quite a quick simple technique that depends on attachment of the CS to the monolith by van der Waals or electrostatic forces [75]. The weakness of these physical forces limits the possibility to use mobile phases that would lead to partial loss of the CS by disruption of those physical forces. In addition, quality assurance about the homogeneity of the CS layer is not guaranteed. To overcome these drawbacks and ensure better homogeneity and more mobile phase versatility, one may use covalent bonding of the CS on the monolith via different linkages (amino [29], ether [76] or triazole [77]) that are most common for introducing CS onto the monoliths. Recently, the advantage of speed, specificity, and large equilibrium constants offered by “click reactions” such as the brush-type copper-catalyzed azide–alkyne cycloaddition reaction [78] have been exploited in the covalent binding of several CS to monoliths such as CDs [79] among others. Immobilization of chiral selectors on epoxy monolithic silica columns is currently attracting increased interest due to some successful results [80]. Figure 1 shows chromatograms for the enantiomeric separation of terbutaline using cellulase as a chiral selector immobilized on epoxy monolithic silica column (Chromolith ^®^ WP 300 Epoxy 100 × 4.6 mm, Merck, Darmstadt, Germany). However, when protein is the CS (BSA [81], OVM [82], and lipase [83] encapsulation has been more frequent due to its simplicity compared to the adsorption of covalent bonding. Yet, leakage of the CS is still a challenge with the encapsulation approach.

## 3. Enantiomeric Impurity Determination

**Figure 2:**
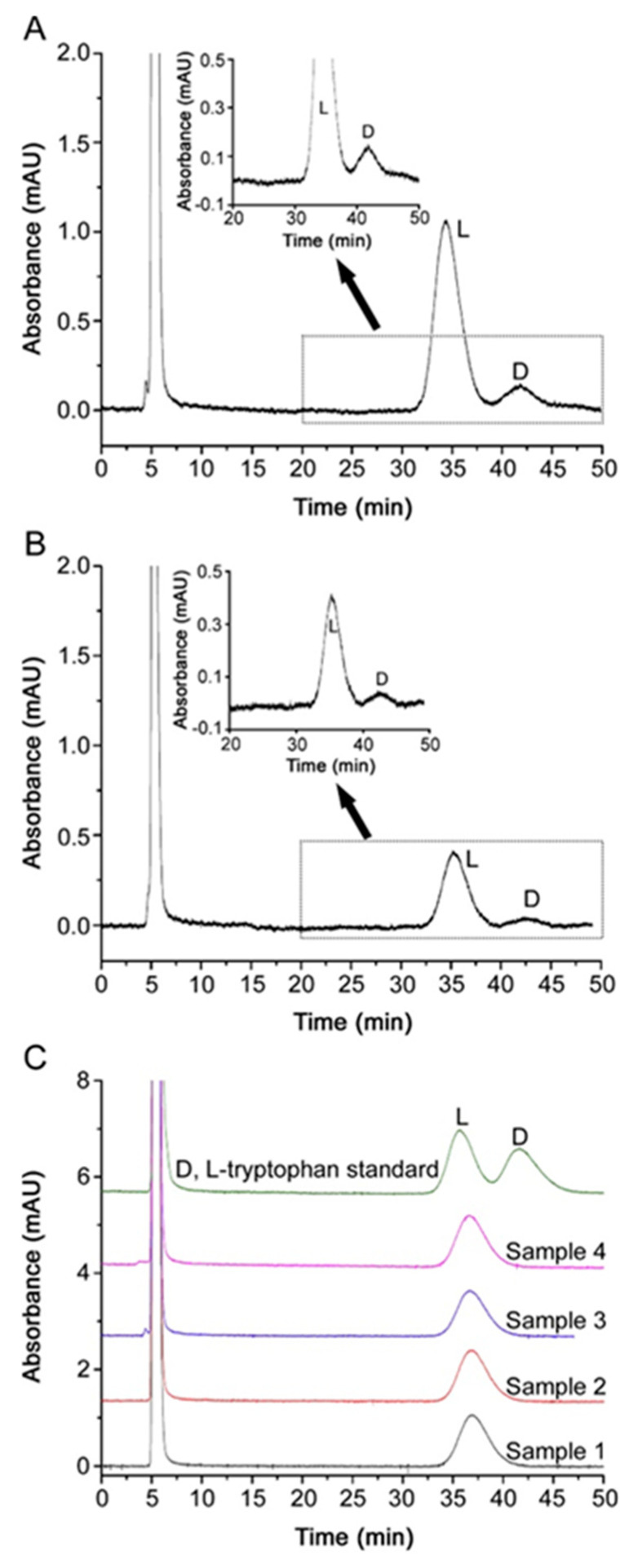
Chromatograms corresponding to (**A**) the LOD (7.5 μM) and (**B**) the LOQ (25 μM) of d-tryptophan; (**C**) FMOC-derivatized racemic tryptophan standard solution and analyzed dietary supplements whose label indicates presence of l-tryptophan (samples 1–4). Ex-perimental conditions: 15 cm × 100 μm I.D.; mobile phase: ACN/3 mM ammonium acetate (65/35, *v/v*) (apparent pH = 4.8); UV detection wavelength: 254 nm; flow rate: 10 μL/min; backpressure: 23 bar; injection volume: 20 nL. Reprinted with permission from reference [90].

Over the years, many different chiral monolithic silica-based HPLC columns have been developed and used to achieve separation of enantiomers [72,84,85]. For instance, an affinity silica-based monolith containing immobilized α1-acid glycoprotein (AGP) was used as chiral stationary phase and evaluated in the chiral separation of R/S-warfarin and R/S-propranolol [86]. Similarly, Mallik & Hage [87] also prepared an affinity monolith based on silica containing immobilized human serum albumin (HSA), which was evaluated in terms of its binding, efficiency and selectivity in the separation of R/S-warfarin, besides the enantioseparation of other chiral compounds, like D/L-tryptophan and R/S-ibuprofen. Likewise, Pittler & Schmid [88] carried out the enantioseparation of dansyl amino acids by HPLC on a monolithic column dynamically coated with a vancomycin derivative. Xu et al. [89], prepared an O-[2-(methacryloyloxy)-ethylcarbamoyl]-10,11-dihydroquinidine (MQD)-silica hybrid monolithic column, which was applied for the chiral analysis of 52 N-derivatized protein and non-protein amino acids. Accordingly, the same research group, achieved the enantioseparation of L-norvaline and L-tryptophan in dietary supplements using an O-[2-(methacryloyloxy)-ethylcarbamoyl]-10,11-dihydroquinidine-silica hybrid monolithic column [90]. However, many of these works are only focused in testing the suitability and chiral discrimination of the stationary phase, without considering the importance of the application itself. In this sense, only some of these works identify and differentiate among the enantiomers [86,87,90], providing the elution order of the active enantiomer and the enantiomeric impurity. This is an important issue to be addressed when performing chiral separations, because the production of enantiomerically pure formulations requires stricter quality controls for pharmaceutical laboratories, as the percentage of the enantiomeric impurity, if present, must be monitored. Accordingly, the International Council for Harmonization (ICH) has set the guidelines for the correct development and validation of analytical methods and the marketing of chiral compounds [91]. In this sense, besides assessing essential analytical parameters, such as linearity, selectivity, matrix effects, accuracy, precision, detection, and quantification limits (LOD and LOQ, respectively), it is of the utmost importance to evaluate the sensitivity of the method. Sensitive methods are required because, according to these ICH guidelines, the enantiomeric impurity cannot exceed a 0.1% of the active enantiomer in the pure enantiomer drug formulation [91]. This is determined by a parameter so-called relative limit of detection (RLOD), which can be calculated as the LOD for the enantiomeric impurity divided by the concentration of the active enantiomer injected × 100, which has to be lower than 0.1% to be sensitive enough, according to the ICH regulation [3,91]. To fulfill this requirement, the most suitable elution order is that the enantiomeric impurity elutes before the active enantiomer. Otherwise, high resolution separation among enantiomers is needed (at least ≥2) so that the peak tailing effect of the active enantiomer, which is a consequence of its high concentration, does not hide the enantiomeric impurity when it is present at a low concentration level (≤0.1%) (Figure 2).

**Figure 3:**
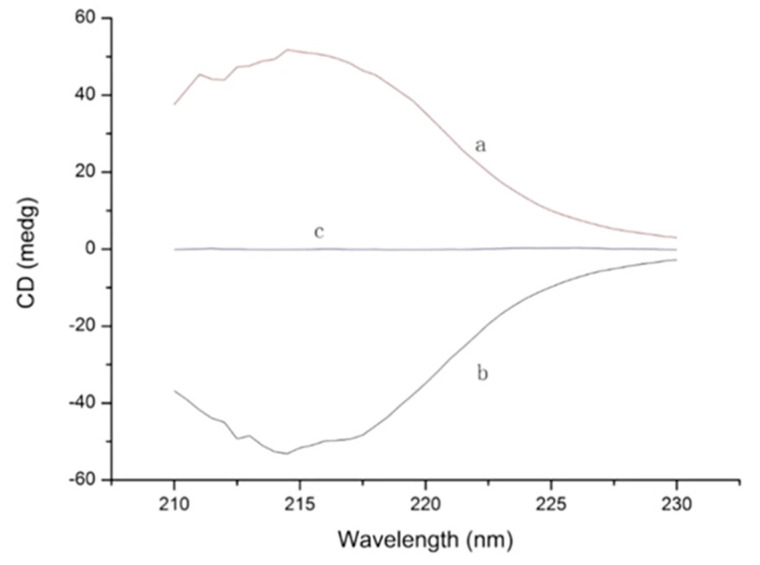
The circular dichroism spectra of L- and D-phenylalanine. (a and b) are the circular dichroism spectra of the same amount of L- and D-phenylalanine, respectively. They have an approximately equal but opposite signal, while (c) is the spectrum from a mixture (racemate) of the same amount of L- and D-phenylalanine. Reprinted with per-mission from reference [95].

Based on this, the identification of enantiomers is an important issue when developing a chiral method. However, sometimes, it can be a challenging task. Enantiomers show the same atomic composition and bonds, so their identification and differentiation with potent detection techniques, such as mass spectrometry or ultraviolet detection, is not possible, because they have the same mass or ultraviolet spectra. For this reason, to properly identify each individual enantiomer and determine the elution order, it is necessary to use pure enantiomer reference products with high purity degree. In this sense, they can be individually injected under chiral conditions, so that the specific retention time of each enantiomer can be determined separately. Nevertheless, sometimes, the acquisition of each individual pure enantiomer standard can be expensive. Thus, an alternative is using a racemic standard and spiking it with one pure enantiomer standard or with the single enantiomer drug formulation (the target sample under evaluation) in order to obtain non-racemic mixtures (for instance, an enantiomeric ratio 1:2). This way, the peak with higher area can be identified as the enantiomer which has been enriched in the racemic mixture. Nevertheless, for chiral compounds with more than one stereogenic centre, more than one pure enantiomer standard will be needed for the identification of all the stereoisomers. On the other hand, sometimes, pure enantiomer standards are commercially unavailable, e.g., in the case of newly developed chiral drugs or multiple chiral centers compounds. In these cases, circular dichroism spectroscopy (CDS) is a powerful tool to assess the absolute configuration of stereoisomers. CDS is a form of light absorption, which measures differences in the absorbance of right- and left- circularly polarized light by compounds. Since enantiomers absorb the portions of light differently, each of them provides a different signal in the circular dichroism spectrum (a pair of enantiomers shows mirrored spectra, i.e., equal but opposite circular dichroism signal), which enables their identification. Other methods have also been used for the identification of enantiomers. For instance, mechanisms of host–guest association, equilibrium ion–molecule reactions, collision induced dissociation of diastereomeric complexes, or ion mobility spectrometry can also be used. Nevertheless, their main problem is that they are limited to iso-tope labeling, specific ligands, or mass spectrometry able to store fragments [92,93]. Indirect methods based on enzymatic digestion for degradation of a specific enantiomer have also been proposed for peak identity of chiral compounds [94]. Nevertheless, the main drawback of this procedure is that not all enantiomers have appropriate digestive enzymes. The electronic circular dichroism can be used to identify the individual enantiomers. Enantiomers will have mirrored circular dichroism spectra, which would be equal but opposite as shown in Figure 3.

## 4. Molecular Modeling Applied to Enantioseparations

Not only is a molecular modeling method needed to elucidate molecular level mechanisms, there is also a demand for using simulation methods to provide the optimal experimental conditions. Many factors in the experiment can affect the separation performance, e.g., the type of chiral stationary phases (CSPs) [96], mobile phases and additives [97], column temperatures [98,99], and the pH conditions [100]. Traditionally, the trail-and-error approach is used to explore the best experimental conditions from various combinations. However, with the understanding of the chiral separation recognition from molecular modeling studies, experiment efforts can be reduced significantly. 

Noting that the majority of this review paper has focused on the monolithic silica-based chiral selector, we shall also notice that most of the up-to-date molecular modeling works exclude the silica support. The main advantage of filling a column with a porous monolithic material is the high permeability of the bed and the low back-pressure generated on the column, which makes it possible to use high mobile phase flow rates which result in shortening the time necessary for the chromatographic run, without a loss of separation efficacy. For enantiomeric separations, the same set of chiral selectors (e.g., polysaccharide-based, cyclodextrin derivatives-based, brush/Pirkle types) used in the conventional particle-packed columns are used in porous monolithic silica. It is also worth mentioning that the pore size on the silica gel is usually larger than the simulation box. So, the simulation on a full-picture HPLC surface is normally beyond the computational capability.

Nevertheless, the most important reason for ruling out the silica support in molecular modeling is that the chiral separation actually takes place at the functional surface, and the separation process is primarily based on the functional chiral stationary phase rather than the silica support. Molecular dynamics simulations for modeling interactions between enantiomers and chiral selectors on silica do not include interactions with the silica support itself. Therefore, the chiral recognition mechanisms will be the same, and the differential interactions of R and S enantiomers with the chiral selectors that are physically adsorbed or covalently bonded to a silica monolith are unchanged from the conventional particle-packed platform. To this end, proper assumptions are then made to account for the role of the monolithic silica support in a chiral separation process: (1) It provides the stationary support where the functional chiral materials are pinned down; (2) It restrains the motion of the chiral functional materials, which allows it to have more interaction with the chiral molecules; (3) The shape of the functional chiral materials is kept relatively immovable. To this end, assuming the stiffness of part of the chiral selector or restraining the motion of the chiral selectors are some popular and reasonable assumptions that have been taken. Such assumptions result in most of the molecular modeling work based on the docking and the molecular dynamics methods. While such approximations provide a viable way to simulate the chiral selector and study the separation mechanism, it is also worth mentioning that the diffusion and adsorption on the porous monolithic silica support could also affect the separation performance. Insights, including enantioselective and non-selective binding energies and adsorption processes, have been investigated by using adsorption isotherms on the chiral stationary phase [101]. Besides, both the immobilization technique and the silica matrix property can affect the chiral selector on the surface, resulting in different separation performances [102]. 

To understand the chiral separation process, the most widely accepted mechanism of the interaction between chiral selectors and enantiomers is often referred to as the Easson–Stedman “three-point interaction” model [103]. However, such a mechanism replies on a static configurational recognition model which fails to account for the fact that the separation is a dynamic process, in which the “static” structure can form, break and reform with respect to time naturally. As mentioned above, “rigidifying” some part of the chiral stationary phase is often the solution to account for the role of the silica support when it is not included in molecular modeling. Although such an assumption results in the loss of dynamic information, it does offer an alternative way to simulate the interaction of a chiral molecule with the chiral stationary phase, and also provides some insights at the molecular level. Using a combined method of NMR and atomistic molecular dynamics (MD) simulation, Ye et al. [104] studied interactions between a 12-mer of amylose tris(3,5-dimethylphenyl carbamate) (ADMPC) with a fixed backbone and the enantiomers. However, their simulation only lasted for 2 ns, which is a relatively short simulation length for such a system. They also assume the fixed backbone structure, which might cause artifacts and affect results. Furthermore, implicit solvent simulations fail to account for the polymer structure relaxation with the presence of solvent molecules. Similar MD studies have been carried out by Kasat et al. [105], focusing on a system with a rigidified polymer backbone structure without solvent molecules. The effect of the solvent has often been neglected in computational studies, which may result in misleading mechanisms for polysaccharide-based CSPs. MD studies on protein binding have shown that the implicit solvent model could lead to significant artifacts in the result [106]. The molecular docking method, which is popular in drug design, also has the similar issue of ignoring the relaxation of the selector structure. Li et al. [107] used molecular docking and MD simulations to study interactions between rigidified polymer selectors and metalaxyl/benalaxyl enantiomers, and they concluded that the formation of hydrogen bonds can affect the separation process.

In a series of studies, Wang et al. adopted a model that consists of a single polymer selector free floating in solutions [108,109]. However, this model neglects the fact that polymers are actually attached on the silica gel instead of free-floating in solutions. Such an on-the-fly model may lead to insufficient sampling between polymer selector and chiral enantiomers. As an improvement, they constructed a more realistic model which included four polymer chains and an amorphous silica slab, which best describes the molecular structure of the CSP surface [110,111,112]. Special cares have been taken to ensure the final model of ADMPC on amorphous silica still maintains the correct polymer structure in the presence of solvent. The goal of the introduction of such silica slab is to pin down and refrain the motion of chiral polymers, so that molecular dynamics can run properly, and information can be sampled adequately. 

**Figure 4:**
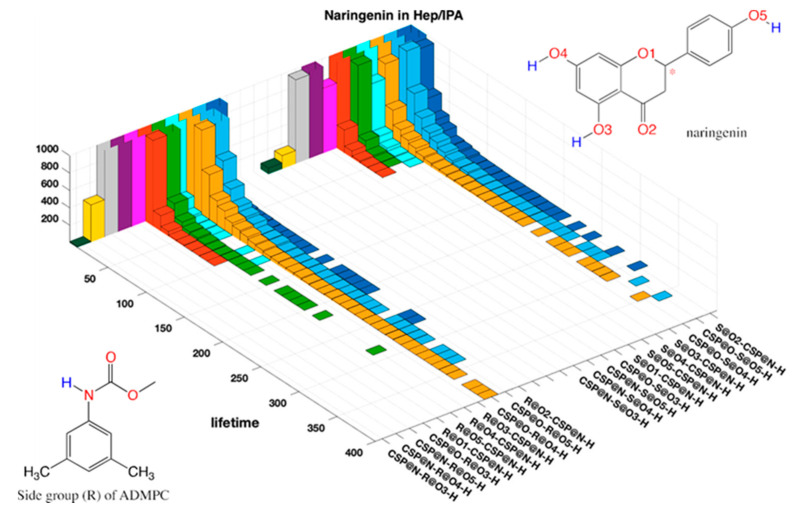
Distribution of lifetimes (ps) of hydrogen bonds between R or S enantiomers of naringenin and ADMPC on silica in heptane/isopropanol 90:10 for each of the acceptor–donor pairs: CSP@O and CSP@N denote the acceptor sites and CSP@N–H denotes the donor site in the chiral stationary phase ADMPC strands. S@O1-5, and R@O1-5 denote the five acceptor sites and S@O3-5H and R@O3-5H denote the three donor sites on the S and R enantiomers of naringenin, respectively. These are the sites identified in top right and bottom left figures for naringenin and ADMPC. The y axis counts the number of incidences over the entire trajectory; the very high counts for the very short lifetimes are cut off in this display. [Adapted with permission from [112]].

It is clear that hydrogen bonding has a major contribution to the interaction between chiral drugs and selectors. Long-lived hydrogen bonds between an enantiomer and the CSP will slow down drug molecules passing through the column, thus resulting in a longer residual time. Wang et al. selected the ADMPC as the chiral selector. The ADMPC has a certain backbone structure which can determine its groove shape on the polymer surface and only allow enantiomers with a certain chirality to sit inside the groove. Such a spatial preference can be indicated by hydrogen bonding features, since preferable structure will allow stronger hydrogen bonding and thus longer lifetime. For instance, Figure 4 shows a hydrogen bonding distribution of naringenin interacting with the ADMPC chiral selector from MD simulations. From Figure 4, the elution order can be concluded from distributions readily, as various donor–acceptor pairs are showing that the (r)-naringenin has more hydrogen bonding structures (proved by higher columns), and longer-lived hydrogen bonding structures (proved be longer tailing columns). Longer lifetimes and higher frequencies of hydrogen bonding events can often induce a longer residual time when drug molecules pass through the HPLC column. Those findings correlate well with the experimental HPLC work which indicates that (r)-naringenin elutes later [113].

**Figure 5:**
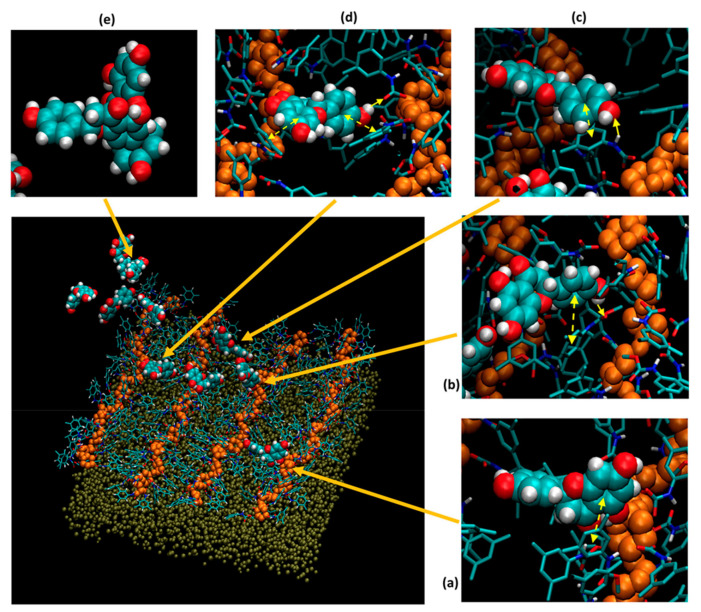
A snapshot of naringenin interacting with ADMPC polymers on amorphous silica slab. Insets (**a**)–(**e**) are zoom-in looks at individual naringenin molecules interacting with the polymer strands, or in a fairly rare event, forming a dimer. [Reprinted with permission from [112]].

Figure 5 shows a particular snapshot of naringenin passing through an ADMPC selector column, when various molecular events happen. A dashed line with double arrows indicates a ring–ring interaction event and a solid line with a single arrow pointing from donor to acceptor indicates a hydrogen bonding event. The most interesting event is perhaps the insert (d) in Figure 5, showing that naringenin can form hydrophobic ring–ring interactions with two polymer selectors simultaneously while a hydrogen bonding structure also appears. Such a molecular structure ensures that hydrogen bonding structure is facilitated by the hydrophobic ring–ring interaction, thus leads to longer-lived lifetime and longer retention time. Note that, although naringenin is mentioned here, it not a unique case. Previous studies have revealed several other drugs, including valsartan, benzoin, and thalidomide, that have similar molecular recognition when passing through a column coated with the ADMPC selector [110,111,112]. 

To conclude, given the fact that the molecular modeling provides reasonably good predictions regarding elution orders against experiments, it is also able to offer molecular level insights, including hydrogen bonding behaviors, ring–ring stacking, and sterical effects, which are critical to understand chiral separation recognition between selectors and drug molecules. While there should be other important molecular events and interactions, modern machine learning technology can help to elucidate and reveal better prediction models.

## Data Availability

The data presented in this study are available within the article or on request from the corresponding author.
